# EXPLORING THE OUTCOME OF DEMENTIA EDUCATION AMONG UNIVERSITY STUDENTS IN TAIWAN

**DOI:** 10.1093/geroni/igae098.2357

**Published:** 2024-12-31

**Authors:** Jouchieh Wang, Tsuann Kuo

**Affiliations:** Chung Shan Medical University, Taichung, Taichung, Taiwan (Republic of China); Chung Shan Medical University, Taichung, Taichung, Taiwan (Republic of China)

## Abstract

Training students to become familiar with dementia is crucial at medical universities as they will most likely to serve people with dementia in the future. This study employed a dementia literacy test and a semi-structured interview to university students to investigate whether a multi-purpose dementia program enhanced students’ sensitivity and attitudes toward dementia-related issues. A total of 415 students participated in pre-arranged dementia lectures, courses, extracurricular activities and service learning. Nine university students were invited to participate in in-depth interviews. The results showed that students had more positive attitudes and knowledge towards dementia. Students were exposed to knowledge-based learning by multi-disciplinary lectures, hands-on skill training and various types of alternative therapies such as arts, music or horticulture. Students felt that they gained empathy, communication skills, and dispelled stereotypes about dementia. After attending various activities within half a year, students not only became familiar with dementia issues but also reflected on what the medical profession can do for people with dementia. Students started to join contests to design dementia activities and to test different programs using design thinking on dementia patients and their caregivers. It was obvious that when students were exposed to various types of dementia activities on campus, the dynamics to learn among peers and to care for dementia family became a culture f on campus. In conclusion, this paper will showcase the multi-purpose training model on dementia which is to be implemented as Taiwan prepares to set its national dementia policy for the next 8 years.

